# Comorbidity burden, management, and in-hospital outcomes in centenarians with proximal hip fracture: a nationwide cohort study (2004–2020)

**DOI:** 10.1007/s11657-025-01576-7

**Published:** 2025-07-11

**Authors:** Juan Carlos Piñeiro-Fernández, Ramón Rabuñal-Rey, Eva Romay-Lema, David Rubal-Bran, Cristina Pedrosa-Fraga, Ana María Santos-Martínez, Yoana Besteiro-Balado, Roi Suárez-Gil, Sonia Pértega-Díaz

**Affiliations:** 1 Internal Medicine Department, Lucus Augusti University Hospital, SERGAS, 1 Ulises Romero Street, 27003 Lugo, Spain; 2 Infectious Diseases Unit, Lucus Augusti University Hospital, SERGAS, 1 Ulises Romero Street, 27003 Lugo, Spain; 3 Department of Health Sciences, Faculty of Nursing and Podiatry, Universidade da Coruña, Rheumatology and Health Research Group, 15403 Ferrol, Esteiro Spain; 4 Instituto de Investigación Biomédica de A Coruña (INIBIC), Nursing and Health Care Research Group, Xubias de Arriba 84, 15006 Coruña, Spain

**Keywords:** Centenarians, Hip fractures, Comorbidity, Mortality, Patient outcome assessment, Electronic health records

## Abstract

**Summary:**

This study analyses comorbidity, surgical management, and complications and their impact on in-hospital outcomes in centenarian hip fracture patients admitted in Spain, 2004–2020. It provides evidence on the prognostic impact of comorbidity and in-hospital complications and highlights the need for specific interventions to improve care in this vulnerable population.

**Purpose:**

This work aims to describe the clinical characteristics, in-hospital progress, and risk factors for worse in-hospital outcomes in centenarian patients with proximal hip fracture (PHF).

**Methods:**

A retrospective nationwide cohort study was conducted that included all centenarian patients hospitalized for PHF (2004–2020) according to the Spanish National Health System’s Minimum Basic Data Set. Demographic, clinical, and hospitalization-related variables were analyzed. Univariate and multivariate analyses were performed.

**Results:**

This study included 4261 patients (83.3% women). The mean Charlson comorbidity index (CCI) was 0.9 ± 1.2; 11.4% had severe comorbidity. Surgery was performed in 87.2% of patients and in 44.5% after 48 h of admission. Higher CCI scores (OR 1.3, 95% CI 1.0–1.7) and admission to medical departments (OR 4.11, 95% CI 3.0–5.6) were associated with nonsurgical management. Surgical delays ≥ 48 h were associated with admissions on Saturdays (OR 1.9, 95% CI 1.3–2.8) or to medical departments (OR 2.79, 95% CI 1.34–5.83) and with the development of ≥ 3 complications (OR 1.5, 95% CI 1.1–2.0). Overall, 15% of patients died during hospitalization, with significantly higher mortality in nonsurgical patients (31.8% vs. 12.5%, *p* < 0.001). In surgical patients, mortality and prolonged hospital stays were primarily related to higher CCI scores and complications.

**Conclusions:**

Centenarians with PHF have a low severe disease burden but high in-hospital mortality risk. Key predictors of mortality in surgical patients include higher CCI scores and in-hospital complications. This highlights the relevance of integrated care and early optimization of clinical status. Prospective studies with long-term follow-up are needed to better characterize prognostic factors.

**Supplementary Information:**

The online version contains supplementary material available at 10.1007/s11657-025-01576-7.

## Introduction

Population aging has led to an overall increase in the incidence of proximal hip fractures (PHF) in older adults [1]. This phenomenon entails a significant burden and economic impact on healthcare systems due to the morbidity associated with PHF, the increase in the number of hospital admissions (and the complications associated with them), as well as the subsequent rehabilitation process [2]. In Spain, the number of hospital admissions due to PHF in individuals over 65 years of age has progressively increased over the last 20 years [3]. In centenarians, this condition has become the third cause of hospital admission [4].

As individuals age, it becomes more difficult to identify variables related to adverse outcomes after PHF due to the complex interaction between non-modifiable factors (such as degree of comorbidity or fracture type) and potentially modifiable factors (such as surgical delay, length of hospital stay (LOS), decompensation of chronic diseases, or development of acute complications) [5]. In a group as vulnerable as centenarians [6], PHF represents a clinical challenge and can have devastating consequences, with a significant increase in complications and high rates of in-hospital mortality [7, 8]. This makes it essential to consider factors that may adversely affect prognosis early in an individual’s admission.

However, there are very few studies in individuals aged 100 years or older that evaluate predictive factors of adverse outcomes related to hospitalization, both preoperatively and postoperatively [9, 10]. Moreover, the findings are controversial due to the low level of evidence and methodological limitations [11]. Recognizing the clinical profile of centenarians with PHF and potentially modifiable factors during hospitalization may help to promote strategies that optimize clinical outcomes in these patients within integrated, multidisciplinary care programs that have been shown to be effective in other age groups, but have not been specifically evaluated in centenarians [12].

The aim of this study is to describe the clinical characteristics of centenarian patients with PHF and their clinical management and to identify factors associated with increased risk of adverse hospital outcomes, paying particular attention to surgical delay, LOS, and mortality.

## Methods

### Data source

This study used a similar methodology to that described in other works by this research group [4]. It is based on a nationwide retrospective cohort study that included all patients aged 100 years or older hospitalized with a principal diagnosis of PHF in all Spanish National Health System (SNS, for its initials in Spanish) hospitals between January 1, 2004, and December 31, 2020. The SNS is a decentralized system comprising the autonomous communities’ health services, which have the capacity to manage their resources autonomously under a common health law. It provides universal healthcare coverage to Spanish citizens [13].

Data were drawn from the Hospital Discharge Records-Minimum Basic Data Set (HDR-MBDS), a mandatory registry for all public and private hospitals in Spain. This dataset, which is universally accessible to any researcher upon request to the Spanish Ministry of Health, includes standardized demographic, clinical, and hospitalization-related variables. In accordance with Spanish legislation, all data are anonymized before being provided to the principal investigator, thus eliminating the need for individual patient consent or ethics committee approval [13].

Patients with a principal diagnosis of PHF were identified using the International Classification of Diseases, Ninth Revision, Clinical Modification (ICD-9-CM) codes 820 (820.0–820.9) up to 2016 and ICD-10-CM codes S72 (S72.0, S72.1, and S72.2) up to 2020. Only emergency admissions were included; planned admissions, PHFs due to complications of previous surgery, high-energy trauma, tumors, and periprosthetic etiology were excluded. Data on each admission described in this study included year of hospitalization, sex, age, admitting department, secondary diagnoses (up to 20), procedures (up to 20), type of management (medical or surgical), date of surgery, type of discharge (home, transfer to a nursing home, transfer to another hospital, or death), and LOS. Delay in surgery was defined as the time in hours from admission to surgery and was divided into three groups (< 24 h, 24–48 h, ≥ 48 h).

Secondary diagnoses and procedures were coded using ICD-9-CM and ICD-10-CM codes. All secondary diagnoses were reviewed to identify chronic conditions and assess patient comorbidity. A total of 32 chronic diseases were included in the analysis, which were grouped into 16 common disease categories based on the ICD-10-CM. Multimorbidity was defined as the presence of two or more chronic diseases in the same individual. The Charlson comorbidity index (CCI), an index adapted for use with administrative databases, was used to quantify comorbidity. The CCI defines severe comorbidity (which has been associated with an annual in-hospital mortality rate of over 50%) as a score greater than two points [14].

PHFs were classified according to injury characteristics as extracapsular, pertrochanteric or subtrochanteric, or intracapsular. The type of surgery was classified as internal fixation (open reduction or closed reduction) and arthroplasty (hemiarthroplasty or total arthroplasty).

The following in-hospital complications were also included: respiratory tract infection (RTI), urinary tract infection (UTI), acute respiratory failure, acute kidney injury (AKI), electrolyte imbalance, functional gastrointestinal disorders, anemia, malnutrition, delirium, pressure ulcers, and pharmacological complications.

Quality control techniques were performed on the database to improve the statistical analysis as well as to simplify the results and their visualization. Variables that occurred with a frequency of less than 3% in the study population and those considered not clinically relevant in the study’s context were excluded.

### Statistical analysis

A descriptive analysis of the characteristics of patients admitted for PHF during the study period was performed. Quantitative variables were expressed as means, standard deviations, and medians, whereas qualitative variables were expressed as frequencies and percentages.

Univariate analysis was performed to compare the characteristics of centenarians with PHF according to sex, surgical decision, and surgical delay. The normality of quantitative variables was assessed using the Kolmogorov–Smirnov test. For continuous variables, Student’s *t*-test or the Mann–Whitney test were used when comparing two groups of patients and the ANOVA or Kruskal–Wallis test when comparing more than two groups. The chi-square or Fisher’s exact test were used for categorical variables. Finally, Spearman’s rank correlation coefficient was used to analyze correlations between continuous variables. A multivariate analysis was then performed. First, a binary logistic regression model was used to analyze factors independently associated with a non-surgical approach, while a multinomial logistic regression analysis was performed to identify characteristics associated with longer surgical delays. The variable of surgical delay (< 24 h, 24–48 h, and ≥ 48 h) was considered, with a delay < 24 h used as a reference.

A competing risks approach was used to estimate the probability of in-hospital death or discharge alive, treating death and hospital discharge as competing events. Univariate and multivariate cause-specific Cox proportional hazards and Fine-Gray regression models were calculated to assess the influence of different variables on either event, as these two methods are considered complementary in the context of competing risks [15]. Based on these analyses, both cause-specific hazard ratios (HRs) and the subdistribution hazard ratios (sHRs) associated with each variable were estimated, along with their 95% confidence intervals (CI). Thus, when hospital discharge was the outcome, an HR or sHR > 1 indicated that the presence of the variable increased the likelihood of being discharged alive, thereby reducing LOS.

All variables significantly associated with each outcome on the univariate analysis as well as those considered to be potential confounders, were included in the multivariate models. All multivariate models were adjusted for a linear effect of year, as significant temporal trends in the management and outcomes of PHF in Spanish centenarians were identified in a previous work by this research group [16].

All analyses were two-tailed and values of *p* < 0.05 were considered significant. Statistical analysis was performed using software IBM SPSS, version 29.0 (IBM, New York City, NY, USA) and RStudio version 2023.06.1, including the cmprsk and mstate packages.

## Results

### Baseline clinical characteristics and sex-related differences in centenarian patients admitted for PHF

Between 2004 and 2020, a total of 4261 admissions of centenarian patients with PHF were recorded in Spanish hospitals. Table 1 shows the characteristics of all episodes included. The mean age was 101.3 ± 1.6 years, and 3549 (83.3%) were women. Only 14.2% of patients had been institutionalized before the PHF, more frequently in women than in men.

**Table 1:** Characteristics of hip fracture admissions in centenarians, 2004–2020, by sex

	Total *n* = 4261	Male *n* = 712 (16.7%)	Female *n* = 3549 (83.3%)	*p*
Age, years	101.3 ± 1.6	101.3 ± 1.7	101.3 ± 1.6	0.574
Injury characteristics				
Intracapsular fracture	1761 (41.3)	370 (52.0)	1391 (39.2)	< 0.001
Pertrochanteric fracture	2228 (52.3)	317 (44.5)	1911 (53.8)	
Subtrochanteric fracture	272 (6.4)	25 (3.5)	247 (7.0)	
Department of admission				
Traumatology	3855 (94.7)	629 (92.8)	3226 (95.1)	0.031
Medical department	215 (5.3)	49 (7.2)	166 (4.9)	
Number of chronic diseases	2.0 ± 1.7	2.0 ± 1.6	2.0 ± 1.6	0.298
0	842 (19.8)	147 (20.6)	695 (19.6)	
1	997 (23.4)	177 (24.9)	820 (23.1)	
≥ 2	2422 (56.8)	388 (54.5)	2034 (57.3)	
CCI	0.9 ± 1.2	1.2 ± 1.5	0.9 ± 1.2	< 0.001
Severe comorbidity, CCI ≥ 3	484 (11.4)	119 (16.7)	365 (10.3)	< 0.001
Comorbidities				
Hypertension	1813 (42.5)	228 (32.0)	1585 (44.7)	< 0.001
Dyslipemia	353 (8.3)	54 (7.6)	299 (8.4)	0.504
Diabetes	416 (9.8)	55 (7.7)	361 (10.2)	0.053
CAD	287 (6.7)	72 (10.1)	215 (6.1)	< 0.001
CHF	519 (12.2)	88 (12.4)	431 (12.1)	0.932
AF	526 (12.3)	102 (14.3)	424 (11.9)	0.089
Bradyarrhythmias	166 (3.9)	38 (5.3)	128 (3.6)	0.038
COPD	273 (6.4)	94 (13.2)	179 (5.0)	< 0.001
CVD	235 (5.5)	51 (7.2)	184 (5.2)	0.043
CKD	517 (12.1)	107 (15.0)	410 (11.6)	0.011
Dementia	587 (13.8)	73 (10.3)	514 (14.5)	0.003
Type of procedure				0.030
No surgery	544 (12.8)	109 (15.3)	435 (12.3)	
Surgery	3717 (87.2)	603 (84.7)	3114 (87.7)	< 0.001
Internal fixation	1970 (64.3)	270 (51.6)	1700 (66.9)	
Open reduction	620 (31.5)	92 (34.1)	528 (31.1)	
Closed reduction	1350 (68.5)	178 (65.9)	1172 (68.9)	
Arthroplasty	1094 (35.7)	253 (48.4)	841 (33.1)	
Surgical delay, days*	2.8 ± 2.6	3.1 ± 2.7	2.8 ± 2.6	0.010
< 24 h	471 (15.5)	57 (11.5)	414 (16.3)	
24–48 h	643 (21.2)	106 (21.5)	537 (21.1)	
> = 48 h	1921 (63.3)	331 (67.0)	1590 (62.6)	
In-hospital complications				
RTI	200 (4.7)	50 (7)	150 (4.2)	0.002
UTI	220 (5.2)	25 (3.5)	195 (5.5)	0.037
Acute respiratory failure	257 (6)	51 (7.2)	206 (5.8)	0.192
AKI	321 (7.5)	58 (8.1)	263 (7.4)	0.548
Hydroelectrolytic disorders	228 (5.4)	43 (6)	185 (5.2)	0.422
Functional gastrointestinal disorders	251 (5.9)	34 (4.8)	217 (6.1)	0.194
Anemia	1157 (27.2)	176 (24.7)	981 (27.6)	0.120
Transfusion	1025 (24.1)	144 (20.2)	881 (24.8)	0.010
Urinary catheterization	167 (3.9)	32 (4.5)	135 (3.8)	0.447
Malnutrition	351 (8.2)	53 (7.4)	298 (8.4)	0.441
Delirium	295 (6.9)	62 (8.7)	233 (6.6)	0.048
Pressure ulcers	185 (4.3)	30 (4.2)	155 (4.4)	0.934
Pharmacological complications	400 (9.4)	74 (10.4)	326 (9.2)	0.348
No. in-hospital complications	1.2 ± 1.5	1.2 ± 1.5	1.2 ± 1.6	0.931
None	2021 (47.4)	335 (47.1)	1686 (47.5)	
1	884 (20.7)	156 (21.9)	728 (20.5)	
2	627 (14.7)	104 (14.6)	523 (14.7)	
≥ 3	729 (17.1)	117 (16.4)	612 (17.2)	
In-hospital mortality	639 (15.0)	122 (17.1)	517 (14.6)	0.080
Place of discharge, nursing home^**^	327 (9.2)	64 (11.1)	263 (8.8)	0.083
LOS, days	10.6 ± 8.6	11.3 ± 8.4	10.5 ± 8.6	0.018
Survivors	10.8 ± 8.6	11.6 ± 8.3	10.6 ± 8.6	
Non-survivors	9.5 ± 8.7	9.7 ± 8.7 (7.5)	9.5 ± 8.6	

Note: Continuous variables are expressed as mean ± standard deviation and categorical variables as number (percentage). *CCI* Charlson comorbidity index, *CAD* coronary artery disease, *CHF* chronic heart failure, *AF* atrial fibrillation, *COPD* chronic obstructive pulmonary disease, *CVD* cerebrovascular disease, *CKD* chronic kidney disease, *RTI* respiratory tract infection, *UTI* urinary tract infection, *AKI* acute kidney injury, *LOS* length of hospital stay. *Surgery date only available in 3035 of surgical cases. **Percentage among those patients discharged alive

Multimorbidity was observed in 56.8% of patients and 11.4% had severe comorbidity, with a mean CCI score of 0.9 ± 1.2. The most prevalent comorbidities were hypertension, dementia, atrial fibrillation (AF), and chronic kidney disease (CKD), with significant differences between the sexes. While women had higher rates of hypertension and dementia, men had higher rates of coronary artery disease (CAD), arrhythmias other than AF, chronic obstructive pulmonary disease (COPD), cerebrovascular disease (CVD), and CKD. Men also had higher CCI scores and a greater proportion of severe comorbidity.

In terms of fracture type, extracapsular fractures accounted for more than half (58.7%) of all episodes. Overall, 94.7% of centenarians were admitted to traumatology and orthopedic surgery departments. According to sex, extracapsular fractures and admission to a traumatology and orthopedic surgery department were significantly more common in women, whereas intracapsular fractures and admission to a medical department were more common in men.

### Factors associated with surgical treatment

A total of 3717 (87.2%) centenarians underwent surgery. These patients were significantly more likely to be female; present with extracapsular fractures; be admitted to a traumatology and orthopedic surgery department; have less comorbidity as measured by the CCI; and have a lower prevalence of cardiovascular disease, COPD, and dementia (Table 2). Internal fixation was the most common surgical procedure, especially in women, while arthroplasty was more frequent in men (Table 1). The characteristics of centenarians with PHF treated surgically according to the type of surgery performed are shown in Supplementary Table 1.

**Table 2:** Characteristics of centenarian patients with hip fracture distributed by the surgical decision

	No surgery *n* = 544	Surgery *n* = 3717	*p*
Age, years	101.5 ± 1.8	101.3 ± 1.6	0.106
Gender, female	435 (80.0)	3,114 (83.8)	0.030
Injury characteristics			< 0.001
Intracapsular fracture	331 (60.8)	1430 (38.5)	
Pertrochanteric fracture	195 (35.8)	2033 (54.7)	
Subtrochanteric fracture	18 (3.3)	254 (6.8)	
Department of admission			< 0.001
Traumatology	441 (85.3)	3414 (96.1)	
Medical department	76 (14.7)	139 (3.9)	
Number of chronic diseases	2.1 ± 1.6 (2)	2.0 ± 1.7 (2)	0.483
0	95 (17.5)	747 (20.1)	
1	135 (24.8)	862 (23.2)	
≥ 2	314 (57.7)	2108 (56.7)	
CCI	1.2 ± 1.4	0.9 ± 1.2	< 0.001
Severe comorbidity, CCI ≥ 3	88 (16.2)	396 (10.7)	< 0.001
Comorbidities			
Hypertension	217 (39.9)	1596 (42.9)	0.195
Dyslipemia	45 (8.3)	308 (8.3)	0.999
Diabetes	55 (10.1)	361 (9.7)	0.830
CAD	48 (8.8)	239 (6.4)	0.047
CHF	89 (16.4)	430 (11.6)	0.002
AF	79 (14.5)	447 (12)	0.113
Bradyarrhythmias	40 (7.4)	126 (3.4)	< 0.001
COPD	49 (9.0)	224 (6.0)	0.011
CVD	41 (7.5)	194 (5.2)	0.035
CKD	78 (14.3)	439 (11.8)	0.106
Dementia	101 (18.6)	486 (13.1)	0.001
In-hospital complications			
RTI	29 (5.3)	171 (4.6)	0.520
UTI	33 (6.1)	187 (5)	0.360
Acute respiratory failure	47 (8.6)	210 (5.6)	0.008
AKI	49 (9)	272 (7.3)	0.191
Hydroelectrolytic disorders	31 (5.7)	107 (5.3)	0.777
Functional gastrointestinal disorders	34 (6.3)	217 (5.8)	0.777
Anemia	89 (16.4)	1068 (28.7)	< 0.001
Transfusion	56 (10.3)	969 (26.1)	< 0.001
Urinary catheterization	17 (3.1)	150 (4)	0.366
Malnutrition	37 (6.8)	314 (8.4)	0.222
Delirium	30 (5.5)	265 (7.1)	0.195
Pressure ulcers	28 (5.1)	157 (4.2)	0.382
Pharmacological complications	31 (5.7)	369 (9.9)	0.002
No. in-hospital complications	0.9 ± 1.4	1.2 ± 1.6	< 0.001
None	278 (51.1)	1743 (46.9)	
1	141 (25.9)	743 (20.0)	
2	67 (12.3)	560 (15.1)	
≥ 3	58 (10.7)	671 (18.1)	
In-hospital mortality	173 (31.8)	466 (12.5)	< 0.001
Place of discharge, nursing home *	25 (6.8)	302 (9.4)	0.105
LOS, days	7.8 ± 11.0	11.0 ± 8.1	< 0.001
Survivors	8.2 ± 11.6	11.1 ± 8.1	
Non-survivors	7.0 ± 9.6	10.5 ± 8.1	

Note: Continuous variables are expressed as mean ± standard deviation and categorical variables as number (percentage). *CCI* Charlson comorbidity index, *CAD* coronary artery disease, *CHF* chronic heart failure, *AF* atrial fibrillation, *COPD* chronic obstructive pulmonary disease, *CVD* cerebrovascular disease, *CKD* chronic kidney disease, *RTI* respiratory tract infection, *UTI* urinary tract infection, *AKI* acute kidney injury, *LOS* length of hospital stay. *Percentage of patients discharged alive

A multivariate logistic regression analysis (Table 3) showed that centenarians with a higher mean CCI score (OR 1.3, 95% CI 1.0–1.7, *p* = 0.040) and those admitted to departments other than a traumatology and orthopedic surgery department (OR 4.11, 95% CI 3.0–5.6, *p* < 0.001) were more likely not to undergo surgical treatment for PHF. Likewise, intracapsular fractures were operated on at a lower rate than subtrochanteric and pertrochanteric fractures (*p* < 0.001). A linear effect was also observed, which confirmed a downward trend in the proportion of patients who did not undergo surgery over the study period (OR 0.97, CI 95% 0.95–0.99, *p* < 0.001).

**Table 3:** Multivariate analysis of characteristics associated with no surgical treatment (logistic regression) or delayed surgery in PHF centenarians (multinomial regression)

	Logistic regression			Multinomial regression (reference group: surgery delay < = 24 h)					
	No surgical treatment			Time-to-surgery 24–48 h			Time-to-surgery ≥ 48 h		
	*p*	OR	95% CI	*p*	OR	95% CI	*p*	OR	95% CI
Gender, female	0.545	0.93	0.73–1.18	0.133	0.76	0.54–1.09	0.071	0.75	0.55–1.02
Injury characteristics	< 0.001								
Intracapsular fracture		1			1			1	
Subtrochanteric fracture	< 0.001	0.33	0.20–0.55	0.189	0.70	0.42–1.19	0.281	0.79	0.51–1.21
Pertrochanteric fracture	< 0.001	0.41	0.33–0.50	0.019	0.73	0.56–0.95	< 0.001	0.58	0.46–0.73
Department of admission (non-Traumatology)	< 0.001	4.11	3.03–5.58	0.019	2.60	1.17–5.77	0.006	2.79	1.34–5.83
CCI	0.040	1.32	1.01–1.72	0.483	1.15	0.78–1.68	0.519	1.11	0.80–1.55
Day of admission									
Monday				0.116	1.44	0.91–2.26	0.194	1.30	0.87–1.94
Tuesday				0.194	1.34	0.86–2.07	0.49	1.15	0.78–1.68
Wednesday					1			1	
Thursday				0.034	1.61	1.04–2.49	0.471	1.16	0.78–1.71
Friday				0.007	0.52	0.32–0.84	0.294	1.22	0.84–1.78
Saturday				0.095	0.65	0.40–1.08	0.002	1.85	1.25–2.75
Sunday				0.227	1.33	0.84–2.09	0.115	1.38	0.92–2.05
Year of admission	0.007	0.97	0.95–0.99	0.326	0.99	0.96–1.02	<.001	0.93	0.91–0.95

*Note: OR* odds ratio, *CI* confidence intervale, *CCI* Charlson comorbidity index

### Factors associated with longer surgical delay

**Fig. 1:**
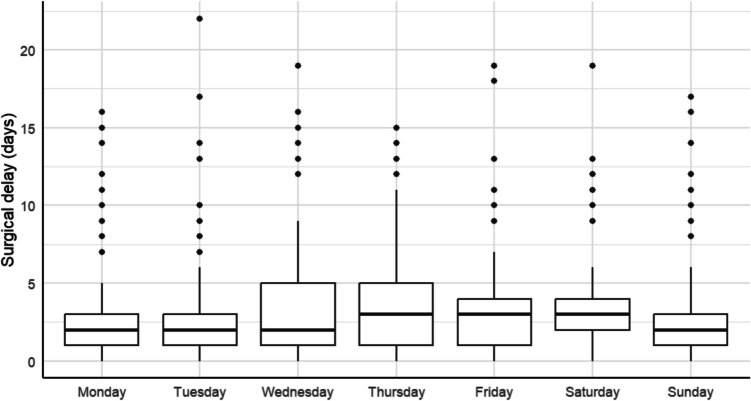
Distribution of time to surgery according to weekday of admission for centenarian patients undergoing hip fracture surgery in Spain, 2004–2020

Surgical delay occurred in 88.9% (3305) of surgical patients. Of these, it was longer than 48 h in 44.5%. The mean surgical delay was 2.8 ± 2.6 days. Patients with longer delays were more likely to be male, have pertrochanteric fractures, and be admitted during the weekend (Fig. 1). Delays were also longer in those who underwent arthroplasty (Supplementary Table 2). No correlation was observed between surgical delay and comorbidity (Spearman’s rank correlation coefficient =  − 0.005, for number of chronic diseases; Spearman’s rank correlation coefficient = 0.016, for CCI), while patients with longer delays in surgery were more likely to have two or more complications during their hospital stay (*p* = 0.044).

The multinomial logistic regression model (Table 3) showed that admission for a PHF on a Thursday significantly increased the odds of a delay of 24–28 h (OR 1.6, 95% CI 1.0–2.5, *p* = 0.034), while admission on a Saturday was significantly associated with a delay ≥ 48 h (OR 1.9, 95% CI 1.3–2.8, *p* = 0.002). Admission to a non-traumatology and orthopedic surgery department was also significantly associated with a longer surgical delay. Conversely, surgical delay was shorter in patients with pertrochanteric fractures (OR 0.7, *p* = 0.019 for a delay of 24–48 h and OR = 0.6, *p* < 0.001 for a delay of ≥ 48 h). A linear association with year of admission was also observed (OR 0.92. 95% CI 0.90–0.95, *p* < 0.001), confirming a temporal trend toward shorter surgical delays that persisted after adjustment for potential confounders. In addition, when the number of in-hospital complications was included in the model (Supplementary Table 3), the development of three or more complications during hospitalization (OR 1.5, 95% CI 1.1–2.0, *p* = 0.011) was also significantly associated with a surgical delay ≥ 48 h.

### Factors associated with in-hospital outcomes after hip-fracture

The most common in-hospital complications were anemia (and associated transfusions), pharmacological complications, malnutrition, AKI, and delirium. According to sex, a significantly higher proportion of men had AKI and delirium, whereas women had higher rates of UTIs and transfusions (Table 1). Regarding the surgical approach, patients who underwent surgery had a higher total number of complications and more episodes of anemia, transfusions, and pharmacological complications, whereas those who did not undergo surgery had more episodes of acute respiratory failure (Table 2).

A total of 639 (15%) centenarian patients died during hospitalization. In-hospital mortality was significantly higher in patients who did not undergo surgery (31.8% vs. 12.5%; *p* < 0.001), but no differences were observed according to sex or surgical delay (Table 1 and Supplementary Table 2). The overall mean LOS was 10.6 ± 8.6 (median LOS was 9 days for survivors and seven for those who died in the hospital). The LOS was significantly longer in men than in women and in patients who underwent surgery compared to those who did not (Tables 1 and 2). In surgical patients, the median postoperative LOS was 7 days (7 days for survivors and 6 for those who died during admission) and was significantly longer in patients with a surgical delay ≥ 48 h (Supplementary Table 2). Supplementary Table 4 shows the mortality and post-surgery LOS according to different characteristics of centenarians with PHF who underwent surgery.

**Fig. 2:**
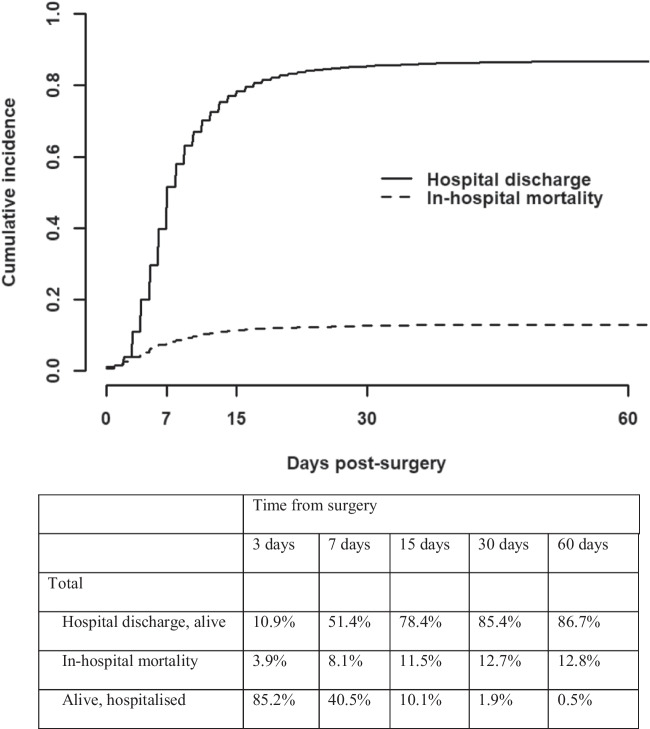
Cumulative incidence function for discharge alive (solid line) or in-hospital death (dashed line), estimated using competing-risk methods, for surgical patients

In addition, Fig. 2 shows the cumulative incidence of in-hospital mortality and those discharged alive among surgical patients with PHF. Of the patients who died, 64.5% died in the first 7 days and 90.4% died in the first 15 days after surgery. More than three-quarters (78.4%) of patients were discharged alive 15 days after surgery. In addition, Supplementary Fig. 1 shows that the cumulative incidence of mortality was significantly higher in patients who did not undergo surgery during the hospitalization.

A univariate analysis of the characteristics associated with in-hospital death and postoperative LOS is shown in Supplementary Table 5. Based on these results, a multivariate analysis was performed (Table 4). The competing risk model identified pertrochanteric fractures and admission to a traumatology and orthopedic surgery department as being significantly associated with the hazard of being discharged alive and, therefore, a shorter LOS. In contrast, higher CCI scores and an increasing number of in-hospital complications were significantly associated with longer LOS. Moreover, a significant trend toward shorter LOS over time was confirmed. In terms of in-hospital mortality, a higher CCI score, and an increasing number of hospital complications were also significantly associated with an increased risk of death.

**Table 4:** Multivariate analysis for time from surgery to discharged alive and time from surgery to in-hospital death for centenarian patients undergoing hip fracture surgery. Cause-specific hazard ratios and sub-distribution hazard ratios obtained from Cox regression model and the Fine-Gray competing risk model, respectively

	**Length of post-surgery hospital stay**					**In-hospital mortality**			
	**HR**	**95% CI**	**sHR**	**95% CI**	**HR**		**95% CI**	**sHR**	**95% CI**
Sex, female	1.043	0.934–1.164	1.028	0.928 1.139	1.044		0.787–1.386	0.97	0.74–1.28
Injury characteristics									
Intracapsular fracture	1		1		1			1	
Subtrochanteric fracture	0.928	0.786–1.097	0.94	0.80–1.11	0.972		0.651–1.45	1.04	0.70–1.55
Pertrochanteric fracture	1.067	0.98–1.162	1.10	1.01–1.19*	0.929		0.742–1.162	0.88	0.71–1.10
Department of admission (Traumatology vs. Other)	2.631	2.132–3.248*	1.53	1.33–1.75*	2.603		1.611–4.204*	1.14	0.74–1.76
CCI	0.976	0.943–1.011	0.95	0.92–0.99*	1.098		1.016–1.187*	1.11	1.04–1.19*
No. in-hospital complications									
None	1		1		1			1	
1	0.858	0.771–0.955*	0.78	0.70–0.87*	1.72		1.278–2.316*	1.92	1.42–2.58*
2	0.785	0.694–0.888*	0.75	0.67–0.84*	1.532		1.098–2.138*	1.85	1.33–2.57*
≥ 3	0.514	0.456–0.58*	0.54	0.48–0.60*	1.701		1.269–2.279*	2.74	2.06–3.63*
Surgical delay, days									
< 24 h	1		1		1			1	
24–48 h	1.134	0.993–1.294	1.01	0.89–1.15	1.227		0.853–1.765	1.16	0.81–1.66
> = 48 h	1.115	0.995–1.25	1.01	0.91–1.13	1.2		0.873–1.65	1.12	0.82–1.53
Year of admission	1.052	1.042–1.062*	1.03	1.02–1.04*	1.03		1.004–1.056*	0.99	0.97–1.02

Note: *CCI* Charlson comorbidity index, *HR* hazard ratio, *sHR* subhazard ratio, *CI* confidence interval; *p < 0.05

## Discussion

This nationwide study describes a combination of clinical, epidemiological, and hospitalization-related outcomes that increase the available evidence on PHF in centenarians, with the largest series described to date. These fractures represent a clinical challenge due to the high vulnerability of centenarians. The findings underscore the importance of recognizing factors associated with increased morbidity and mortality in order to improve decision making and care models in this special group.

Centenarians have different clinical and epidemiological characteristics than octogenarians and nonagenarians, which may influence the management and hospital outcomes of PHFs [17, 18]. Centenarians patients can be considered autonomous but not healthy [6]. Although they present a lower rate of severe comorbidity (as measured by the CCI) and better functional capacity [19, 20], recent studies have shown a progressive accumulation of chronic conditions [21] and they have a high frailty index [22]. This frailty makes them particularly vulnerable to complications after a stressful event such as PHF [23], a condition that determines a significantly worse evolution and prognosis than centenarians without PHF, with higher rates of complications, significant functional deterioration, and, in general, less favorable health outcomes [4, 11].

The findings of this study are in line with previous studies in patients aged 100 years or older with PHF [8, 9, 19, 24], with a clinical profile characterized by a low prevalence of severe comorbidity and limiting chronic diseases (cancer, CAD, CVD, or COPD), at a much lower rates than that described in octogenarians or nonagenarians [18, 20], in contrast to a high prevalence of common chronic diseases (hypertension, CHF, CKD, or AF), with rates similar to those in nonagenarians [19]. In contrast, the proportion of patients with dementia was lower than reported in other series (13.8% vs. 30–50%) [17, 25, 26], possibly due to methodological differences and the inherent limitations of administrative databases, where underreporting and undercoding of secondary diagnoses are frequent, and the recording of stratifiable variables (such as dementia) is particularly challenging in older adults populations [11, 25]. Similarly, the proportion of patients who were institutionalized prior to hospital admission was lower than that reported in previous studies conducted in Spain [17, 26]. This discrepancy can be explained by methodological differences and by the heterogeneity of social and healthcare models and policies in the Spanish autonomous communities [4]. In addition, the study confirms a higher prevalence of extracapsular fractures and an incidence predominantly in women, findings consistent with the literature. This may be explained by the increase in extracapsular fractures with age and the higher risk of falls and functional dependence in centenarian females compared to centenarian males [7, 17, 18].

Despite the ethical dilemmas involved in decision-making in patients with PHF at much older ages and the increasing clinical complexity of centenarians [27], surgical treatment should be prioritized for several reasons: it is a protective factor of survival [10] and is cost-effective in this age group [28]; conservative treatment, particularly in cases with more comorbidity, leads to early morbidity and mortality [26]; and the goal of surgery is to improve functionality and relieve pain regardless of age, vital prognosis, or degree of comorbidity [29]. Although this study confirms that surgery is the predominant treatment, it found a greater proportion of patients who did not undergo surgery compared to similar studies [17, 26], mainly in those admitted to medical departments and in those with a higher degree of comorbidity. This finding highlights the potential for improvements in care to reduce barriers to accessing surgery, even in the most complex patients [7, 30].

Another common dilemma is determining the best time for surgery, as this is a key factor in hospital outcomes. Although intervention within the first 48 h is recommended [31], the evidence is heterogeneous in older adult patients due to the greater difficulty in assessing the impact of surgical delay on clinical outcomes and identifying the factors associated with them [1, 29]. In line with previous works [19, 32], this study does not find an association between surgical delay and mortality but does find an association with a delay of more than 48 h and postoperative LOS. This study also did not demonstrate an association with comorbidity or male sex, in contrast to previous research on centenarians and octogenarians or nonagenarians [20, 31].

This lack of a relationship between comorbidity and prolonged surgical delay could be explained by the clinical profile of centenarians, characterized by lower severity but accompanied by a perception of greater surgical risk due to extreme frailty, which could prioritize surgery to minimize complications [7, 10, 25]. Although globally, PHFs are more frequent in women and more severe in men (who have a longer surgical delay, as demonstrated in this study, due to a higher surgical risk profile and comorbidity) [33, 34], it is possible that this correlation loses significance among centenarians due to a certain gender bias. Although this study identifies a weekend effect (longer delays in patients admitted on Saturdays), this association is not uniform in the literature, which shows discordant results due to organizational factors specific to each health system [30, 35]. Finally, in line with expectations, the delay was shorter in patients admitted to traumatology and orthopedic surgery departments and in those with pertrochanteric fractures, which highlights the influence of the care model and type of fracture on surgical times [29].

In-hospital complications are common in cases of PHF and some of them, such as delirium or acute respiratory failure, have been associated with a significant reduction in survival [36, 37]. However, there have been very limited cases documented in centenarians and, in general, the available studies record complication rates similar to those described in this work and lower than in younger cohorts [19, 24, 29]. A relevant finding in this series is the low proportion of patients diagnosed with delirium (6.9%), considerably lower than the 30% reported in both nonagenarians and other centenarian cohorts [18, 37]. This discrepancy is most likely due to the underdiagnosis and undercoding of acute complications, particularly delirium, in administrative databases.

This work confirms that complications affect more than half of the patients and that they can influence the outcomes as well as the prognosis of the surgical group. In this surgical group, the presence of a high number of complications (especially three or more) is associated with greater delay, longer postoperative LOS, and mortality. The development of complications and a higher degree of comorbidity (as measured by the CCI) were found to be the main predictors of prolonged LOS and lower survival. Previous literature supports these findings, establishing that comorbidity is the main predictor of complications (which are independently associated with lower survival) and mortality across the age spectrum of patients with PHF [1, 18, 38].

Notably, many of these complications are potentially predictable, preventable, and treatable if addressed proactively and early [5, 12]. This is particularly relevant in centenarians, a group in which the risk of mortality after PHF is two to four times higher than in younger populations [8, 11, 19] and with an in-hospital mortality rate of 10%–15% [26, 29] that is especially concentrated in the acute period, and in nonsurgical patients [18], in line with the results of this study.

Based on the aforementioned outcomes, the importance can be deduced of prioritizing admission to traumatology and orthopedic surgery departments over other departments and surgical treatment more so than the specific time at which it is performed, as well as optimizing the health status of patients with more comorbidity before the intervention and at the earliest possible time after it in order to reduce complications and improve the clinical outcome [1, 10, 30, 35]. The care model that has shown the most evidence in achieving these objectives is comanagement. This model intensifies efforts to form multidisciplinary teams and standardize care planning based on the assessment of comorbidities. In addition, it improves care outcomes at all levels by reducing complications through early identification and demonstrates a positive impact on both survival and quality of life in patients with PHF [12, 39].

The main strengths of this work are that it is based on a long-term population-based cohort that reliably represents centenarian patients admitted for PHF in Spain and that it has a very large sample size, which confers great statistical power in the analysis of some variables. In addition, the data were extracted from hospital discharge records in a primary source of clinical information (administrative, official, standardized, and representing practically all Spanish hospitals), with a high degree of external validity and proven use in biomedical research. However, these sources are not specifically designed for research purposes and the data quality depends on the coding accuracy. There may be errors in recording data and there is an important limitation due to the lack of significant clinical information in the context of the study, such as the functional status of patients at admission and discharge, the date of complications (which would allow establishing a temporal sequence between their onset and important outcomes such as surgical delay, LOS, or mortality), medium- and long-term progress, laboratory results, treatments, or the cause of in-hospital death. Future research with prospective studies that include long-term follow-up could contribute to developing early and effective interventions to decrease adverse outcomes among centenarians with PHF, which will increase in the coming years.

## Conclusion

This study confirms that although centenarians with PHF have a low burden of severe disease, they are at high risk of in-hospital mortality. Furthermore, it shows that in patients undergoing surgery, the main predictors of mortality are a greater CCI score and a high number of in-hospital complications, whereas LOS and prolonged surgical delay had no effect on mortality. It also shows some opportunities for improving care to minimize the barriers to accessing surgery and promoting earlier surgery. These findings reinforce the importance of a comprehensive care approach focused on the assessment of comorbidity and early optimization of clinical status in order to prevent complications and improve prognosis. Prospective studies with long-term follow-up are needed to more precisely define prognostic factors and evaluate the impact of multidisciplinary strategies on these patients’ progress.

## Supplementary Information

Below is the link to the electronic supplementary material.

Supplementary file1 (DOCX 519 KB)
