# Supplementary material for: Comorbidity burden, management, and in-hospital outcomes in centenarians with proximal hip fracture: a nationwide cohort study (2004–2020)
Source: Arch Osteoporos. 2025 Jul 11;20(1):88. doi: 10.1007/s11657-025-01576-7 (PMC12254154; doi:10.1007/s11657-025-01576-7)
Supplement: Supplementary file 1 — Supplementary file1 (DOCX 519 KB) [file 11657_2025_1576_MOESM1_ESM.docx]

**SUPPLEMENTARY MATERIAL**

**Supplementary Table 1.** Characteristics of centenarian patients with hip fracture according to the type of surgery.

|  | Internal fixation | | | | | | Arthroplasty | | | | | | | |
| --- | --- | --- | --- | --- | --- | --- | --- | --- | --- | --- | --- | --- | --- | --- |
|  | Open + closed reduction | | Open reduction | | Closed reduction | | Hemi + Total arthroplasty | | Hemiarthroplasty | | | Total arthroplasty | | |
| Age, years | 101.3 ± 1.6 | 101 | 101.2 ± 1.5 | 101 | 101.3 ± 1.6 | 101 | 101.2 ± 1.5 | 101 | 101.2 ± 1.5 | 101 | 101.3 ± 1.5 | | 101 |  |
| Gender, female | 1,700 | 86.3% | 528 | 85.2% | 1,172 | 86.8% | 841 | 76.9% | 746 | 77.7% | 80 | | 72.1% |  |
| Injury characteristics |  |  |  |  |  |  |  |  |  |  |  | |  |  |
| Intracapsular fracture | 264 | 13.4% | 106 | 17.1% | 158 | 11.7% | 1.064 | 97.3% | 935 | 97.4% | 106 | | 95.5% |  |
| Pertrochanteric fracture | 1511 | 76.7% | 447 | 72.1% | 1,064 | 78.8% | 29 | 2.7% | 25 | 2.6% | 4 | | 3.6% |  |
| Subtrochanteric fracture | 195 | 9.9% | 67 | 10.8% | 128 | 9.5% | 1 | 0.1% | 0 | 0.0% | 1 | | 0.9% |  |
| Department of admission |  |  |  |  |  |  |  |  |  |  |  | |  |  |
| Traumatology | 1,782 | 96.6% | 543 | 96.4% | 1,239 | 96.6% | 1,014 | 95.9% | 890 | 96.1% | 101 | | 93.5% |  |
| Medical department | 63 | 3% | 20 | 4% | 43 | 3% | 43 | 4% | 36 | 4% | 7 | | 7% |  |
| Number of chronic diseases | 1.9 ± 1.6 | 2 | 1.8 ± 1.5 | 1 | 1.9 ± 1.6 | 2 | 2.0 ± 1.7 | 2 | 2.0 ± 1.7 | 2 | 2.0 ± 1.6 | | 2 |  |
| 0 | 421 | 21.4% | 144 | 23.2% | 277 | 20.5% | 232 | 21.2% | 207 | 21.6% | 19 | | 17.1% |  |
| 1 | 483 | 24.5% | 169 | 27.3% | 314 | 23.3% | 259 | 23.7% | 221 | 23.0% | 34 | | 30.6% |  |
| >2 | 1,066 | 54.1% | 307 | 49.5% | 759 | 56.2% | 603 | 55.1% | 216 | 22.5% | 22 | | 19.8% |  |
| CCI | 0.8 ± 1.2 | 0 | 0.8 ± 1.2 | 0 | 0.8 ± 1.1 | 0 | 0.9 ± 1.2 | 0 | 0.9 ± 1.2 | 0 | 1.0 ± 1.3 | | 1.0 |  |
| Severe comorbidity, CCI ≥ 3 | 174 | 8.8% | 61 | 9.8% | 113 | 8.4% | 129 | 11.8% | 112 | 11.7% | 15 | | 13.5% |  |
| Comorbidities |  |  |  |  |  |  |  |  |  |  |  | |  |  |
| Hypertension | 838 | 42.5% | 263 | 42.4% | 575 | 42.6% | 481 | 44.0% | 425 | 44.3% | 43 | | 38.7% |  |
| Dyslipemia | 130 | 6.6% | 42 | 6.8% | 88 | 6.5% | 87 | 8.0% | 72 | 7.5% | 13 | | 11.7% |  |
| Diabetes | 185 | 9.4% | 59 | 9.5% | 126 | 9.3% | 103 | 9.4% | 92 | 9.6% | 7 | | 6.3% |  |
| CAD | 203 | 10.3% | 59 | 9.5% | 144 | 10.7% | 131 | 12.0% | 118 | 12.3% | 13 | | 11.7% |  |
| CHF | 125 | 6.3% | 35 | 5.6% | 90 | 6.7% | 71 | 6.5% | 59 | 6.1% | 8 | | 7.2% |  |
| AF | 212 | 10.8% | 64 | 10.3% | 148 | 11.0% | 132 | 12.1% | 119 | 12.4% | 12 | | 10.8% |  |
| Bradyarrhythmias | 69 | 3.5% | 29 | 4.7% | 40 | 3.0% | 26 | 2.4% | 25 | 2.6% | 1 | | 0.9% |  |
| COPD | 102 | 5.2% | 42 | 6.8% | 60 | 4.4% | 75 | 6.9% | 64 | 6.7% | 9 | | 8.1% |  |
| CVD | 86 | 4.4% | 20 | 3.2% | 66 | 4.9% | 66 | 6.0% | 60 | 6.3% | 6 | | 5.4% |  |
| CKD | 191 | 9.7% | 61 | 9.8% | 130 | 9.6% | 131 | 12.0% | 111 | 11.6% | 20 | | 18.0% |  |
| Dementia | 235 | 11.9% | 72 | 11.6% | 163 | 12.1% | 140 | 12.8% | 121 | 12.6% | 18 | | 16.2% |  |
| Surgical delay, days* | 2.9 ± 2.8 | 2 | 3.1 ± 2.9 | 2.0 | 2.9 ± 2.0 | 3 | 3.0 ± 2.5 | 2.0 | 3.0 ± 2.5 | 2 | 2.9 ± 2.5 | | 3 |  |
| < 24 hours | 247 | 16.4% | 71 | 15.5% | 176 | 16.9% | 95 | 10.7% | 81 | 10.3% | 14 | | 13.3% |  |
| 24-48 hours | 304 | 20.2% | 96 | 20.9% | 208 | 19.9% | 189 | 21.2% | 169 | 21.5% | 20 | | 19.0% |  |
| 48 hours – 3 days | 261 | 17.4% | 76 | 16.6% | 185 | 17.7% | 177 | 19.9% | 160 | 20.4% | 17 | | 16.2% |  |
| 3-4 days | 200 | 13.3% | 50 | 10.9% | 150 | 14.4% | 128 | 14.4% | 108 | 13.7% | 20 | | 19.0% |  |
| 4-5 days | 173 | 11.5% | 54 | 11.8% | 119 | 11.4% | 109 | 12.2% | 93 | 11.8% | 16 | | 15.2% |  |
| 5-6 days | 108 | 7.2% | 45 | 9.8% | 63 | 6.0% | 62 | 7.0% | 56 | 7.1% | 6 | | 5.7% |  |
| 6-7 days | 64 | 4.3% | 16 | 3.5% | 48 | 4.6% | 48 | 5.4% | 46 | 5.9% | 2 | | 1.9% |  |
| ≥ 7 days | 145 | 9.7% | 51 | 11.1% | 94 | 9.0% | 83 | 9.3% | 73 | 9.3% | 10 | | 9.5% |  |
| In-hospital complications |  |  |  |  |  |  |  |  |  |  |  | |  |  |
| RTI | 84 | 4.3% | 24 | 3.9% | 60 | 4.4% | 44 | 4.0% | 38 | 4.0% | 6 | | 5.4% |  |
| UTI | 85 | 4.3% | 32 | 5.2% | 53 | 3.9% | 58 | 5.3% | 45 | 4.7% | 12 | | 10.8% |  |
| Acute respiratory failure | 93 | 4.7% | 34 | 5.5% | 59 | 4.4% | 68 | 6.2% | 59 | 6.1% | 9 | | 8.1% |  |
| AKI | 123 | 6.2% | 39 | 6.3% | 84 | 6.2% | 83 | 7.6% | 70 | 7.3% | 11 | | 9.9% |  |
| Hydroelectrolytic disorders | 91 | 4.6% | 25 | 4.0% | 66 | 4.9% | 65 | 5.9% | 49 | 5.1% | 16 | | 14.4% |  |
| Functional gastrointestinal disorders | 108 | 5.5% | 30 | 4.8% | 78 | 5.8% | 65 | 5.9% | 59 | 6.1% | 6 | | 5.4% |  |
| Anemia | 557 | 28.3% | 159 | 25.6% | 398 | 29.5% | 272 | 24.9% | 239 | 24.9% | 29 | | 26.1% |  |
| Transfusion | 517 | 26.2% | 152 | 24.5% | 365 | 27.0% | 240 | 21.9% | 217 | 22.6% | 16 | | 14.4% |  |
| Urinary catheterization | 81 | 4.1% | 27 | 4.4% | 54 | 4.0% | 38 | 3.5% | 33 | 3.4% | 4 | | 3.6% |  |
| Malnutrition | 125 | 6.3% | 28 | 4.5% | 97 | 7.2% | 98 | 9.0% | 87 | 9.1% | 11 | | 9.9% |  |
| Delirium | 113 | 5.7% | 30 | 4.8% | 83 | 6.1% | 77 | 7.0% | 64 | 6.7% | 13 | | 11.7% |  |
| Pressure ulcers | 73 | 3.7% | 20 | 3.2% | 53 | 3.9% | 53 | 4.8% | 44 | 4.6% | 7 | | 6.3% |  |
| Pharmacological complications | 179 | 9.1% | 58 | 9.4% | 121 | 9.0% | 120 | 11.0% | 107 | 11.1% | 10 | | 9.0% |  |
| In-hospital mortality | 238 | 12.1% | 79 | 12.7% | 159 | 11.8% | 137 | 12.5% | 121 | 12.6% | 15 | | 13.5% |  |
| LOS, days | 11.2 ± 8.9 | 10.0 | 11.8 ± 11.5 | 10.0 | 11.0 ± 7.4 | 9.0 | 11.4 ± 7.0 | 10.0 | 11.2 ± 6.5 | 10.0 | 13.2 ± 9.5 | | 11.0 |  |
| Survivors | 11.3 ± 8.9 | 10.0 | 11.6 ± 6.9 | 10.0 | 12.0 ± 11.9 | 10.0 | 10.9 ± 7.2 | 9.0 | 11.3 ± 6.1 | 10.0 | 13.7 ± 9.9 | | 11.0 |  |
| Non-survivors | 11.0 ± 8.4 | 9.0 | 10.3 ± 8.0 | 9.0 | 10.4 ± 7.2 | 9.0 | 11.3 ± 9.0 | 9.0 | 10.4 ± 8.4 | 9.0 | 9.9 ± 4.9 | | 9.0 |  |

Note: Continuous variables are expressed as mean ± standard deviation and categorical variables as number or percentage. CCI = Charlson Comorbidity index; CAD = coronary artery disease; CHF = chronic heart failure; AF = atrial fibrillation; COPD = chronic obstructive pulmonary disease; CVD = cerebrovascular disease; CKD = chronic kidney disease; RTI = respiratory tract infection; UTI = urinary tract infection; AKI = Acute kidney injury; LOS = length of hospital stay. * Surgery date only available in 2393 of surgical cases.

**Supplementary Table 2.** Characteristics of centenarian patients undergoing hip fracture surgery according to surgical delay from admission.

|  | Time-to-surgery | | | | | | | | *p* |
| --- | --- | --- | --- | --- | --- | --- | --- | --- | --- |
|  | < 24 hours | | 24-48 hours | | | ≥ 48 hours | | |  |
| Age, years | 101.4 ± 1.6 | 101 | 101.4 ± 1.7 | 101 | 101.2 ± 1.6 | | 101 | 0.134 | |
| Gender, female | 414 | 87.9% | 537 | 83.5% | 1,590 | | 82.8% | 0.026 | |
| Injury characteristics |  |  |  |  |  | |  | <0.001 | |
| Intracapsular fracture | 140 | 29.7% | 238 | 37.0% | 773 | | 40.2% |  | |
| Pertrochanteric fracture | 299 | 63.5% | 364 | 56.6% | 999 | | 52.0% |  | |
| Subtrochanteric fracture | 32 | 6.8% | 41 | 6.4% | 149 | | 7.8% |  | |
| Day of the week of admission |  |  |  |  |  | |  | <0.001 | |
| Monday | 62 | 13.2% | 108 | 16.8% | 252 | | 13.1% |  | |
| Tuesday | 71 | 15.1% | 119 | 18.5% | 274 | | 14.3% |  | |
| Wednesday | 71 | 15.1% | 87 | 13.5% | 233 | | 12.1% |  | |
| Thursday | 67 | 14.2% | 133 | 20.7% | 250 | | 13.0% |  | |
| Friday | 78 | 16.6% | 52 | 8.1% | 307 | | 16.0% |  | |
| Saturday | 61 | 13.0% | 47 | 7.3% | 345 | | 18.0% |  | |
| Sunday | 61 | 13.0% | 97 | 15.1% | 260 | | 13.5% |  | |
| Department of admission, traumatology | 450 | 98.3% | 595 | 95.4% | 1,738 | | 95.0% | 0.009 | |
| Number of chronic diseases | 1.8 ± 1.5 | 2 | 2.1 ± 1.8 | 2 | 2.1 ± 1.7 | | 2 | 0.105 | |
| 0 | 96 | 20.4% | 136 | 21.2% | 385 | | 20.0% |  | |
| 1 | 126 | 26.8% | 146 | 22.7% | 437 | | 22.7% |  | |
| ≥ 2 | 249 | 52.9% | 361 | 56.1% | 1,099 | | 57.2% |  | |
| CCI | 0.9 ± 1.3 | 0 | 0.9 ± 1.2 | 2 | 2.1 ± 1.7 | | 2 | 0.683 | |
| Severe comorbidity, CCI ≥ 3 | 49 | 10.4% | 78 | 12.1% | 205 | | 10.7% | 0.544 | |
| Comorbidities |  |  |  |  |  | |  |  | |
| Hypertension | 202 | 42.9% | 274 | 42.6% | 798 | | 41.5% | 0.812 | |
| Dyslipemia | 35 | 7.4% | 63 | 9.8% | 166 | | 8.6% | 0.379 | |
| Diabetes | 39 | 8.3% | 61 | 9.5% | 188 | | 9.8% | 0.607 | |
| CAD | 25 | 5.3% | 41 | 6.4% | 125 | | 6.5% | 0.628 | |
| CHF | 51 | 10.8% | 78 | 12.1% | 231 | | 12.0% | 0.750 | |
| AF | 39 | 8.3% | 77 | 12.0% | 257 | | 13.4% | 0.010 | |
| Bradyarrhythmias | 16 | 3.4% | 22 | 3.4% | 71 | | 3.7% | 0.920 | |
| COPD | 28 | 5.9% | 44 | 6.8% | 108 | | 5.6% | 0.525 | |
| CVD | 23 | 4.9% | 43 | 6.7% | 108 | | 5.6% | 0.415 | |
| CKD | 60 | 12.7% | 77 | 12.0% | 239 | | 12.4% | 0.923 | |
| Dementia | 58 | 12.3% | 97 | 15.1% | 246 | | 12.8% | 0.276 | |
| Type of surgery |  |  |  |  |  | |  | <0.001 | |
| Internal fixation | 247 | 72.2% | 304 | 61.7% | 951 | | 61.0% |  | |
| Open reduction | 71 | 28.7% | 96 | 31.6% | 292 | | 30.7% |  | |
| Closed reduction | 176 | 71.3% | 208 | 68.4% | 659 | | 69.3% |  | |
| Arthroplasty | 95 | 27.8% | 189 | 38.3% | 607 | | 39.0% |  | |
| Total arthroplasty | 81 | 85.3% | 169 | 89.4% | 536 | | 88.3% |  | |
| Hemiarthroplasty | 14 | 14.7% | 20 | 10.6% | 71 | | 11.7% |  | |
| In-hospital complications |  |  |  |  |  | |  |  | |
| RTI | 20 | 4.2% | 38 | 5.9% | 91 | | 4.7% | 0.378 | |
| UTI | 17 | 3.6% | 30 | 4.7% | 107 | | 5.6% | 0.192 | |
| Acute respiratory failure | 21 | 4.5% | 41 | 6.4% | 119 | | 6.2% | 0.320 | |
| AKI | 34 | 7.2% | 48 | 7.5% | 148 | | 7.7% | 0.931 | |
| Hydroelectrolytic disorders | 15 | 3.2% | 34 | 5.3% | 113 | | 5.9% | 0.065 | |
| Functional gastrointestinal disorders | 26 | 5.5% | 45 | 7.0% | 115 | | 6.0% | 0.544 | |
| Anemia | 117 | 24.8% | 170 | 26.4% | 558 | | 29.0% | 0.127 | |
| Transfusion | 101 | 21.4% | 170 | 26.4% | 505 | | 26.3% | 0.083 | |
| Urinary catheterization | 16 | 3.4% | 20 | 3.1% | 93 | | 4.8% | 0.103 | |
| Malnutrition | 35 | 7.4% | 62 | 9.6% | 190 | | 9.9% | 0.264 | |
| Delirium | 36 | 7.6% | 51 | 7.9% | 141 | | 7.3% | 0.880 | |
| Pressure ulcers | 15 | 3.20% | 33 | 5.1% | 86 | | 4.5% | 0.288 | |
| Pharmacological complications | 44 | 9.3% | 56 | 8.7% | 222 | | 11.6% | 0.080 | |
| No. in-hospital complications | 1.1 ± 1.4 | 0 | 1.2 ± 1.6 | 1 | 1.3 ± 1.7 | | 1 | 0,044 | |
| None | 242 | 51.4% | 30 | 47.4% | 878 | | 45.7% |  | |
| 1 | 89 | 18.9% | 130 | 20.2% | 392 | | 20.4% |  | |
| 2 | 65 | 13.8% | 85 | 13.2% | 278 | | 14.5% |  | |
| ≥ 3 | 75 | 15.9% | 123 | 19.1% | 373 | | 19.4% |  | |
| In-hospital mortality | 49 | 10.4% | 81 | 12.6% | 245 | | 12.8% | 0.373 | |
| Place of discharge, nursing home* | 27 | 5.8% | 41 | 6.4% | 106 | | 5.5% | 0.711 | |
| Post-surgery stay, days | 8.0 ± 5.9 | 6 | 7.9 ± 6.9 | 6 | 8.4 ± 7.3 | | 7 | 0.101 | |
| Survivors | 8.0 ± 5.8 | 6 | 8.0 ± 6.8 | 6.5 | 8.5 ± 7.1 | | 7 | 0.049 | |
| Non-survivors | 7.9 ± 6.5 | 6 | 6.8 ± 5.8 | 5 | 7.8 ± 8.4 | | 6 | 0.578 | |

*Note*: Continuous variables are expressed as mean ± standard deviation and categorical variables as number or percentage. CCI = Charlson Comorbidity index; CAD = coronary artery disease; CHF = chronic heart failure; AF = atrial fibrillation; COPD = chronic obstructive pulmonary disease; CVD = cerebrovascular disease; CKD = chronic kidney disease; RTI = respiratory tract infection; UTI = urinary tract infection; AKI = Acute kidney injury. * Percentage of patients discharged alive

**Supplementary Table 3.** Results from multinomial logistic regression model describing factors associated with later times from hospital admission to surgery (reference group: surgery delay<=24 hours).

|  | Time-to-surgery 24-48 hours | | | | | Time-to-surgery ≥ 48 hours | | | | |
| --- | --- | --- | --- | --- | --- | --- | --- | --- | --- | --- |
|  | *p* | OR | 95% CI | | *p* | | OR | 95% CI | |  |
| Gender, female | 0.142 | 1.305 | 0.915 | 1.861 | 0.080 | | 1.319 | 0.967 | 1.8 |  |
| Injury characteristics |  |  |  |  |  | |  |  |  |  |
| Intracapsular fracture |  | 1 |  |  |  | | 1 |  |  |  |
| Subtrochanteric fracture | 0.158 | 0.686 | 0.407 | 1.158 | 0.207 | | 0.757 | 0.49 | 1.167 |  |
| Pertrochanteric fracture | 0.015 | 0.718 | 0.55 | 0.938 | <0.001 | | 0.567 | 0.451 | 0.714 |  |
| Department of admission, Traumatology | 0.036 | 0.421 | 0.188 | 0.944 | 0.018 | | 0.409 | 0.195 | 0.859 |  |
| CCI | 0.830 | 1.011 | 0.911 | 1.122 | 0.580 | | 1.026 | 0.938 | 1.122 |  |
| Day of admission |  |  |  |  |  | |  |  |  |  |
| Monday | 0.110 | 1.446 | 0.92 | 2.273 | 0.178 | | 1.315 | 0.883 | 1.959 |  |
| Tuesday | 0.197 | 1.336 | 0.861 | 2.072 | 0.485 | | 1.148 | 0.78 | 1.689 |  |
| Wednesday |  | 1 |  |  |  | | 1 |  |  |  |
| Thursday | 0.029 | 1.629 | 1.051 | 2.526 | 0.424 | | 1.173 | 0.793 | 1.736 |  |
| Friday | 0.007 | 0.516 | 0.319 | 0.836 | 0.309 | | 1.216 | 0.834 | 1.772 |  |
| Saturday | 0.093 | 0.65 | 0.393 | 1.074 | 0.002 | | 1.846 | 1.244 | 2.74 |  |
| Sunday | 0.232 | 1.321 | 0.837 | 2.086 | 0.121 | | 1.371 | 0.92 | 2.042 |  |
| Year of admission | 0.245 | 0.983 | 0.954 | 1.012 | <0.001 | | 0.924 | 0.901 | 0.948 |  |
| No. in-hospital complications |  |  |  |  |  | |  |  |  |  |
| None |  | 1 |  |  |  | | 1 |  |  |  |
| 1 | 0.246 | 1.216 | 0.874 | 1.693 | 0.092 | | 1.277 | 0.961 | 1.698 |  |
| 2 | 0.792 | 1.052 | 0.722 | 1.533 | 0.186 | | 1.24 | 0.902 | 1.704 |  |
| ≥ 3 | 0.092 | 1.36 | 0.951 | 1.944 | 0.011 | | 1.491 | 1.096 | 2.028 |  |

*Note*: OR = odds ratio; CI = confidence intervale; CCI = Charlson comorbidity index.

**Supplementary Table 4.** Mortality and length of postoperative hospital stay in centenarian patients undergoing hip fracture surgery, according to different variables.

|  |  | Mortality  n = 639 | | Length of stay post-surgery | | | |
| --- | --- | --- | --- | --- | --- | --- | --- |
|  |  |  |  | Alive | | Dead | |
|  |  | n | % | Mean ± SD | Median | Mean ± SD | Median |
| Gender | Male | 122 | 20.0% | 9.1±7.4 | 7 | 7.5±8.5 | 6 |
|  | Female | 517 | 15.8% | 8.2±6.7 | 7 | 7.6±7.5 | 6 |
| Department of admission | Other than Traumatology | 44 | 26.2% | 20.7±17.4 | 17 | 20.7±16.8 | 16.5 |
|  | Traumatology | 556 | 15.6% | 7.8±5.4 | 7 | 6.9±6.1 | 5 |
| Injury characteristics | Subtrochanteric fracture | 47 | 22.1% | 9.2±7.1 | 7 | 6.8±7.5 | 6 |
|  | Pertrochanteric fracture | 334 | 16.5% | 8.1±6.8 | 7 | 8.1±7.9 | 6 |
|  | Intracapsular fracture | 258 | 16.4% | 8.4±6.8 | 7 | 7.1±7.4 | 5 |
| Type of surgery | Internal fixation | 238 | 13.6% | 8.6±7.1 | 7 | 8.3±8.3 | 6 |
|  | Arthroplasty | 137 | 14.6% | 8.6±6.3 | 7 | 7.1±7.4 | 5 |
| Type of internal fixation | Open reduction | 79 | 15.5% | 8.9±7.6 | 7 | 7.4±6.7 | 6 |
|  | Close reduction | 159 | 13.6% | 8.4±6.9 | 7 | 8.7±8.8 | 6 |
| Type of arthroplasty | Hemiarthroplasty | 121 | 14.8% | 8.3±5.5 | 7 | 7.0±7.7 | 5 |
|  | Total arthroplasty | 15 | 20.8% | 10.7±10.0 | 8 | 7.3±5.0 | 8 |
| Multimorbidity | No | 252 | 15.3% | 8.1±6.3 | 7 | 6.6±7.3 | 5 |
|  | Yes | 387 | 17.5% | 8.5±7.3 | 7 | 8.2±7.9 | 6 |
| Severe comorbidity | No | 520 | 14.9% | 8.3±6.8 | 7 | 7.6±8.2 | 5 |
|  | Yes | 119 | 28.6% | 8.9±6.8 | 7 | 7.5±5.2 | 6 |
| Comorbidities |  |  |  |  |  |  |  |
| Hypertension | No | 384 | 17.2% | 8.6±7.0 | 7 | 7.4±7.1 | 6 |
|  | Yes | 255 | 15.7% | 8.0±6.7 | 7 | 7.9±8.6 | 5 |
| Dyslipemia | No | 592 | 16.3% | 8.5±7.0 | 7 | 7.6±7.8 | 6 |
|  | Yes | 47 | 17.2% | 7.1±4.5 | 6 | 6.8±5.7 | 5 |
| Diabetes | No | 566 | 15.9% | 8.4±7.0 | 7 | 7.6±7.8 | 6 |
|  | Yes | 73 | 21.4% | 7.4±4.7 | 6 | 7.7±6.7 | 6 |
| CAD | No | 576 | 15.6% | 8.3±6.7 | 7 | 7.6±7.8 | 6 |
|  | Yes | 63 | 27.0% | 9.0±9.2 | 7 | 7.0±6.3 | 4.5 |
| CHF | No | 497 | 14.4% | 8.0±6.3 | 7 | 7.2±7.7 | 5 |
|  | Yes | 142 | 31.3% | 11.3±9.7 | 8 | 8.9±7.5 | 7 |
| AF | No | 534 | 15.4% | 8.2±6.6 | 7 | 7.6±7.9 | 5.5 |
|  | Yes | 105 | 23.5% | 9.3±8.5 | 7 | 7.6±6.8 | 6 |
| Bradyarrhythmias | No | 595 | 15.6% | 8.3±6.7 | 7 | 7.7±7.8 | 6 |
|  | Yes | 44 | 33.6% | 10.0±10.2 | 7 | 4.8±4.1 | 4 |
| COPD | No | 575 | 15.5% | 8.3±6.9 | 7 | 7.7±7.9 | 6 |
|  | Yes | 64 | 28.7% | 9.1±6.4 | 7 | 6.0±4.7 | 4 |
| CVD | No | 599 | 16.0% | 8.4±6.9 | 7 | 7.6±7.8 | 6 |
|  | Yes | 40 | 22.2% | 7.4±4.9 | 6 | 7.7±5.3 | 8 |
| CKD | No | 539 | 15.5% | 8.3±6.9 | 7 | 7.8±8.1 | 5.5 |
|  | Yes | 100 | 22.9% | 8.8±6.2 | 7 | 6.7±4.7 | 6 |
| Dementia | No | 550 | 16.2% | 8.4±6.8 | 7 | 7.7±7.9 | 6 |
|  | Yes | 89 | 18.2% | 7.8±6.9 | 6 | 6.7±6.4 | 5 |
| In-hospital complications |  |  |  |  |  |  |  |
| RTI | No | 560 | 14.9% | 8.1±6.4 | 7 | 7.1±6.9 | 5 |
|  | Yes | 79 | 46.4% | 15.4±12.1 | 12 | 10.5±11.3 | 7 |
| UTI | No | 606 | 16.1% | 8.2±6.7 | 7 | 7.5±7.7 | 6 |
|  | Yes | 33 | 20.2% | 11.6±8.2 | 10 | 9.6±8.0 | 7 |
| Acute respiratory failure | No | 529 | 14.3% | 8.1±6.5 | 7 | 7.3±7.1 | 5 |
|  | Yes | 110 | 48.9% | 13.5±11.1 | 10 | 9.1±10.1 | 6 |
| Hydroelectrolytic disorders | No | 574 | 15.3% | 8.1±6.3 | 7 | 7.5±7.9 | 5 |
|  | Yes | 65 | 34.6% | 13.5±13.5 | 10 | 8.5±5.5 | 8 |
| Functional gastrointestinal disorders | No | 590 | 15.8% | 8.2±6.7 | 7 | 7.3±7.6 | 5 |
|  | Yes | 49 | 24.8% | 10.8±8.3 | 8 | 10.2±7.8 | 8.5 |
| Anemia | No | 458 | 16.0% | 8.0±6.3 | 7 | 7.3±7.3 | 5 |
|  | Yes | 181 | 17.8% | 9.3±8.1 | 7 | 8.1±8.4 | 6 |
| Transfusion | No | 469 | 15.7% | 7.9±6.3 | 7 | 7.2±7.3 | 5 |
|  | Yes | 170 | 19.0% | 9.6±8.1 | 7 | 8.3±8.3 | 6 |
| Uninary chaterization | No | 608 | 16.0% | 8.2±6.6 | 7 | 7.4±7.2 | 5 |
|  | Yes | 31 | 25.0% | 11.4±10.7 | 8.5 | 10.1±13.2 | 8 |
| Malnutrition | No | 607 | 16.7% | 8.1±6.4 | 7 | 7.0±6.1 | 5 |
|  | Yes | 32 | 12.5% | 10.8±9.9 | 8 | 14.6±17.3 | 8 |
| Delirium | No | 588 | 16.0% | 8.2±6.8 | 7 | 7.3±7.0 | 6 |
|  | Yes | 51 | 21.9% | 10.1±7.7 | 8 | 10.7±12.9 | 6 |
| Pressure ulcers | No | 618 | 16.3% | 8.2±6.6 | 7 | 7.4±7.7 | 6 |
|  | Yes | 21 | 16.5% | 12.3±10.2 | 9 | 10.7±6.7 | 12 |
| Pharmacological complications | No | 525 | 14.7% | 8.0±6.2 | 7 | 7.6±7.5 | 6 |
|  | Yes | 114 | 33.1% | 11.7±11.1 | 8 | 7.5±8.2 | 5 |
| No. in-hospital complications | None | 209 | 11.7% | 7.3±5.2 | 6 | 7.1±6.4 | 6 |
|  | 1 | 158 | 20.5% | 8.1±5.7 | 7 | 6.4±6.5 | 4 |
|  | 2 | 112 | 21.0% | 8.7±7.6 | 7 | 6.4±6.8 | 5 |
|  | ≥ 3 | 160 | 25.1% | 11.5±9.9 | 9 | 9.5±9.4 | 7 |

Note: SD = standard deviation; CCI = Charlson Comorbidity index; CAD = coronary artery disease; CHF = chronic heart failure; AF = atrial fibrillation; COPD = chronic obstructive pulmonary disease; CVD = cerebrovascular disease; CKD = chronic kidney disease; RTI = respiratory tract infection; UTI = urinary tract infection; AKI = Acute kidney injury.

**Supplementary Figure 1.** Cumulative incidence function for discharge alive (solid line) or in-hospital death (dashed line), estimated using competing-risk methods, according to treatment approach.


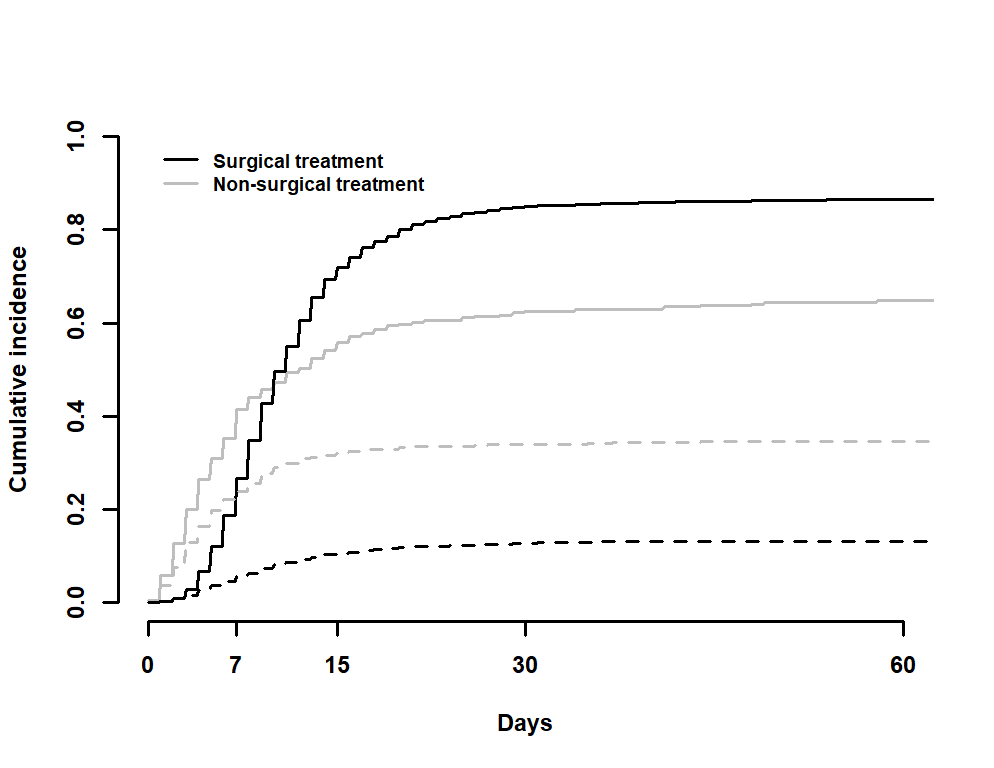


|  | Time from hospital admission | | | | | |
| --- | --- | --- | --- | --- | --- | --- |
|  | 3 days | 7 days | 15 days | 30 days | 60 days |  |
| Total |  |  |  |  |  |  |
| Hospital discharge | 4.9% | 28.4% | 69.9% | 82.2% | 83.8% |  |
| In-hospital mortality | 2.9% | 7.7% | 13.2% | 15.3% | 15.7% |  |
| Alive, hospitalised | 92.2% | 63.9% | 16.9% | 2.5% | 0.5% |  |
| Non-surgical treatment |  |  |  |  |  |  |
| Hospital discharge | 20.0% | 41.3% | 55.8% | 62.5% | 64.8% |  |
| In-hospital mortality | 12.8% | 23.9% | 31.9% | 33.9% | 34.7% |  |
| Alive, hospitalised | 67.2% | 34.8% | 12.3% | 3.6% | 0.5% |  |
| Surgical treatment |  |  |  |  |  |  |
| Hospital discharge | 2.8% | 26.6% | 71.8% | 84.9% | 86.4% |  |
| In-hospital mortality | 1.5% | 5.5% | 10.6% | 12.7% | 13.1% |  |
| Alive, hospitalised | 95.7% | 67.9% | 17.6% | 2.4% | 0.5% |  |

**Supplementary Table 5.** Univariate analysis for time from surgery to discharge and time from surgery to in-hospital death for centenarian patients undergoing hip fracture surgery. Cause-specific hazard ratios and sub-distribution hazard ratios obtained from Cox regression model and the Fine-Gray competing risk model, respectively.

|  | Length of post-surgery hospital stay | | In-hospital mortality | |
| --- | --- | --- | --- | --- |
|  | HR (95% CI) | sHR (95% CI) | HR (95% CI) | sHR (95% CI) |
| Age, years | 1.00 (0,98-1.03) | 0.99 (0.97-1.02) | 1.05 (0.99-1.11) | 1.05 (0.99-1.11) |
| Gender, female | 1.13 (1.01-1.25)* | 1.1 (0.91-1.22) | 0,97 (0,74-1,26) | 0.87 (0.67-1.13) |
| Injury characteristics |  |  |  |  |
| Intracapsular fracture | 1 | 1 | 1 | 1 |
| Subtrochanteric fracture | 0.87 (0.74-1.02) | 0.88 (0.76-1.04) | 1.04 (0.68-1.48) | 1.11 (0.75-1.64) |
| Pertrochanteric fracture | 1.04 (0.96-1.13) | 1.06 (0.98-1.15) | 0.95 (0.77-1.18) | 0.93 (0.75-1.15) |
| Department of admission, traumatology | 3.10 (2.52-3.82)* | 1.86 (1.64-2.12)* | 2.34 (1.45-3.77)* | 0.80 (0.52-1.23) |
| Type of surgery, arthroplasty vs. internal fixation | 0.99 (0.90-1.08) | 0.95 (0.87-1.03) | 1.14 (0.90-1.44) | 1.14 (0.88-1.44) |
| Type of internal fixation, closed vs. open reduction | 1.06 (0.94-1.19) | 1.04 (0.93-1.16) | 1.15 (0.82-1.60) | 1.09 (0.78-1.52) |
| Type of arthroplasty, total vs. hemiarthroplasty | 0.78 (0.62-0.97)* | 0.90 (0.74-1.1) | 0.76 (0.43-1.37) | 0.95 (0.54-1.69) |
| Surgical delay, days |  |  |  |  |
| <24 hours | 1 | 1 | 1 | 1 |
| 24-48 hours | 1.01 (0.89-1.15) | 0.97 (0.85-1.09) | 1.23 (0.87-1.77) | 1.21 (0.85-1.72) |
| > 48 hours | 0.92 (0.83-1.03) | 0.90 (0.81-1.00) | 1.17 (0.86-1.59) | 1.23 (0.91-1.67) |
| Number of chronic diseases | 0.96 (0.94-0.98)* | 0.96 (0.94-0.98)* | 1.04 (0.99-1.11) | 1.08 (1.03-1.14)* |
| Multimorbidity | 0.90 (0.84-0.98)* | 0.91 (0.85-0.98)* | 1.18 (0.96-1.46) | 1.28 (1.04-1.57)* |
| CCI | 0.95 (0.92-0.98)* | 0.92 (0.89-0.95)* | 1.17 (1.10-1.26)* | 1.2 (1.12-1.27)* |
| Severe comorbidity, CCI ≥ 3 | 0.82 (0.72-0.93)* | 0.74 (0.65-0.84)* | 1.18 (1.40-2.35)* | 2.01 (1.56-2.59)* |
| Comorbidities |  |  |  |  |
| Hypertension | 1.09 (1.01-1.19) | 1.1 (1.02-1.18)* | 0.92 (0.75-1.14) | 0.87 (0.71-1.07) |
| Dyslipemia | 1.27 (1.10-1.45)* | 1.19 (1.04-1.36)* | 1.06 (0.72-1.55) | 0.88 (0.60-1.29) |
| Diabetes | 1.12 (0.98-1.28) | 1.03 (0.90-1.18) | 1.30 (0.94-1.79) | 1.17 (0.85-1.61) |
| CAD | 0.90 (0.76-1.06) | 0.83 (0.71-0.98)* | 1.53 (1.08-2.16)* | 1.65 (1.17-2.32)* |
| CHF | 0.59 (0.52-0.68)* | 0.61 (0.54-0.68)* | 1.52 (1.18-1.95)* | 2.19 (1.73-2.79)* |
| AF | 0.83 (0.73-0.93)* | 0.81 (0.73-0.91)* | 1.25 (0.95-1.65) | 1.42 (1.08-1.86)* |
| Bradyarrhythmias | 0.85 (0.69-1.06) | 0.83 (0.68-1.01) | 1.21 (0.75-1.95) | 1.38 (0.86-2.24) |
| COPD | 0.86 (0.72-1.02) | 0.79 (0.67-0.93)* | 1.42 (0.99-2.05) | 1.56 (1.09-2.25)* |
| CVD | 1.11 (0.93-1.31) | 1.04 (0.88-1.24) | 1.25 (0.83-1.90) | 1.13 (0.75-1.7) |
| CKD | 0.89 (0.79-1.01) | 0.85 (0.76-0.95)* | 1.32 (1.01-1.74)* | 1.4 (1.07-1.84)* |
| Dementia | 1.10 (0.98-1.23) | 1.02 (0.91-1.14) | 1.31 (0.99-1.73) | 1.22 (0.92-1.61) |
| In-hospital complications |  |  |  |  |
| RTI | 0.40 (0.32-0.49)* | 0.42 (0.35-0.50)* | 1.70 (1.25-2.32)* | 3.16 (2.37-4.21)* |
| UTI | 0.66 (0.55-0.79)* | 0.74 (0.64-0.85)* | 0.81 (0.53-1.25) | 1.15 (0.75-1.75) |
| Acute respiratory failure | 0.47 (0.39-0.57)* | 0.44 (0.373-0.52)* | 2.21 (1.68-2.90)* | 3.67 (2.83-4.75)* |
| AKI | 0.58 (0.49-0.68)* | 0.54 (0.47-0.62)* | 1.83 (1.40-2.41)* | 2.64 (2.02-3.44)* |
| Hydroelectrolytic disorders | 0.52 (0.43-0.63)* | 0.54 (0.46-0.64)* | 1.49 (1.07-2.05)* | 2.33 (1.71-3.17)* |
| Functional gastrointestinal disorders | 0.67 (0.57-0.79)* | 0.73 (0.63-0.84)* | 1.12 (0.78-1.59) | 1.52 (1.08-2.14)* |
| Anemia | 0.82 (0.75-0.90)* | 0.84 (0.77-0.91)* | 1.14 (0.92-1.42) | 1.34 (1.08-1.65)* |
| Transfusion | 0.77 (0.71-0.85)* | 0.79 (0.73-0.86)* | 1.15 (0.92-1.42) | 1.39 (1.12-1.73)* |
| Urinary catheterization | 0.71 (0.58-0.86)* | 0.77 (0.65-0.91)* | 0.99 (0.64-1.55) | 1.33 (0.87-2.05) |
| Malnutrition | 0.73 (0.64-0.84)* | 0.90 (0.81-1) | 0.53 (0.36-0.78)* | 0.72 (0.49-1.05)* |
| Delirium | 0.74 (0.64-0.86)* | 0.81 (0.71-0.93)* | 0.89 (0.62-1.28) | 1.15 (0.80-1.64) |
| Pressure ulcers | 0.62 (0.51-0.75)* | 0.74 (0.65-0.86)* | 0.62 (0.38-1.03) | 0.93 (0.57-1.52) |
| Pharmacological complications | 0.60 (0.52-0.69)* | 0.54 (0.47-0.61)* | 2.15 (1.70-2.73)* | 3.04 (2.41-3.83)* |
| No. in-hospital complications | 0.85 (0.83-0.88)* | 0.86 (0.84-0.88)* | 1.09 (1.03-1.14)* | 1.22 (1.17-1.28)* |
| None | 1 | 1 | 1 | 1 |
| 1 | 0.83 (0.75-0.92)* | 0.76 (0.68-0.84)* | 1.83 (1.38-2.43)* | 2.09 (1.57-2.77)* |
| 2 | 0.78 (0.69-0.88)* | 0.73 (0.65-0.82)* | 1.66 (1.21-2.28)* | 1.98 (1.44-2.72)* |
| ≥ 3 | 0.52 (0.46-0.58)* | 0.53 (0.48-0.58)* | 1.88 (1.44-2.47)* | 2.98 (2.30-3.86)* |

*Note*: HR = hazart ratio; sHR = subhazard ratio; CI = confidence interval. CCI = Charlson Comorbidity index; CAD = coronary artery disease; CHF = chronic heart failure; AF = atrial fibrillation; COPD = chronic obstructive pulmonary disease; CVD = cerebrovascular disease; CKD = chronic kidney disease; RTI = respiratory tract infection; UTI = urinary tract infection; AKI = Acute kidney injury. * Significant at p<0.05.
